# Ingested (Oral) Adrenocorticotropic Hormone Inhibits IL-17 in the Central Nervous System in the Mouse Model of Multiple Sclerosis and Experimental Autoimmune Encephalomyelitis

**DOI:** 10.4049/immunohorizons.2200023

**Published:** 2022-07-22

**Authors:** Landon J. Dittel, Bonnie N. Dittel, Staley A. Brod

**Affiliations:** * Department of Neurology, Medical College of Wisconsin, Milwaukee, WI; † Versiti Blood Research Institute, Medical College of Wisconsin, Milwaukee, WI; ‡ Department of Microbiology and Immunology, Medical College of Wisconsin, Milwaukee, WI

## Abstract

Experimental autoimmune encephalomyelitis (EAE) is an inflammatory autoimmune disease of the CNS that resembles multiple sclerosis and provides a useful animal model for the evaluation of mechanisms of action for potential immunomodulatory therapies. We have previously shown that oral adrenocorticotropic hormone (ACTH) decreased IL-17 in the gut lamina propria and the spleen and increased CD4^+^ Foxp3^+^ T regulatory cells and IL-10 in the spleen during EAE in the C57BL/6 mouse. However, we did not investigate the specific cellular alterations of proinflammatory and anti-inflammatory factors in the CNS. The aim was to determine if oral ACTH would have a similar clinical effect on inflammatory cytokines in the gut and define specific cellular effects in the CNS in an alternative strain of mice. SJL/J mice were immunized with proteolipid protein peptide 138–151 and gavaged with scrambled ACTH (scrambled α-melanocyte-stimulating hormone) or ACTH 1–39 during ongoing disease. Ingested (oral) ACTH attenuated ongoing clinical EAE disease, decreased IL-6 production, and increased T regulatory cells in the lamina propria and decreased CD4^+^ and γδ IL-17 production in the CNS. Ingested ACTH attenuated EAE clinical disease by decreasing IL-6 in the gut-associated lymphoid tissue and decreasing IL-17 in the CNS. *ImmunoHorizons*, 2022, 6: 497–506.

## INTRODUCTION

Experimental autoimmune encephalomyelitis (EAE) is a T cell–mediated inflammatory autoimmune disease of the CNS that resembles in some aspect the human demyelinating disease multiple sclerosis (MS) (1) and provides a useful animal model for the evaluation of potential therapies for autoimmune diseases (2–4). Inoculation of SJL/J mice with immunogenic peptides from proteolipid protein (PLP), a protein expressed by oligodendrocytes composing the myelin sheath, can activate pathogenic neuroantigen-specific Th17 cells in vivo and produce inflammation in EAE (5).

Class II–restricted CD4^+^ Th cells have been separated into three major nonoverlapping subsets according to their IL gene secretion (6–10). Th1 secrete their signature cytokine IFN-γ, regulating delayed-type hypersensitivity responses and inflammation in EAE (9, 10). The Th2 subset produces the anti-inflammatory cytokines IL-4, IL-10, and IL-13 that inhibit EAE (6, 7, 10). Th17 produces IL-17 and IL-21 that can contribute to autoimmunity (8, 11), including EAE (12, 13). Th1 and Th17 overlap and are both encephalitogenic, with Th17 cells typically losing IL-17 expression in the CNS and thereby producing IFN-γ (14).

Th17 cells are constitutively present throughout the gut-associated lymphoid tissue (GALT); in particular, the intestinal lamina propria (LP) and Peyer’s patches of the small intestine (15). Pathogenic Th17 differentiation is initiated by IL-1 (16), IL-6 (17), and TGF-β (18) and reinforced by IL-23 (19) during the development of IL-17–producing cells originating in the GALT (20). We have previously shown that oral adrenocorticotropic hormone (ACTH) decreased IL-17 levels in the LP and spleen and increased CD4^+^Foxp3^+^ T regulatory (T_reg_) cells and IL-10 in the spleen during in EAE in C57BL/6 (B6) mice (21). In antecedent experiments, oral ACTH also decreased clinical EAE scores and the secretion of IFN-γ (22). In neither of those studies were we able to describe more fully the cellular alterations of proinflammatory and anti-inflammatory factors in the CNS. In addition, it is not known if oral ACTH would be clinically effective in another strain of mouse other than the B6 and whether it would have a similar effect on inflammatory and counterregulatory cytokines seen previously.

In humans, 120 IU ACTH by mouth decreased IL-1 and IL-17 in mitogen-stimulated human PBMCs (23). To continue those studies in humans and explore the mechanism of oral ACTH using adaptive statistical protocols, funding agencies have suggested that we confirm that oral ACTH has activity in at least two mouse strains. If oral ACTH shows effectiveness in other mouse strains, even if the mechanism of action (MOA) of oral ACTH is somewhat different, this finding would provide sufficient data for additional human trials.

We therefore examined whether oral ACTH could inhibit EAE in the SJL/J mouse strain used as a model of relapsing and remitting EAE with distinct genetics from B6 mice (24). Our study focused on frequency of Th17-producing cells and the numbers of T_reg_ cells. In particular, we determined that oral ACTH reduced both CD4^+^ and γδ T cell IL-17 production in the CNS and increased the frequency of CD4^+^ T_reg_ cells in both the LP and CNS.

## MATERIALS AND METHODS

### Induction of active EAE

SJL/J 6–8-wk-old females (The Jackson Laboratory, Bar Harbor, ME) were actively immunized with PLP 139–151, but otherwise maintained, handled, and surveilled as outlined previously (25). Briefly, mice were actively immunized by s.c. injection of 0.2 ml inoculum containing 10–30 μg PLP 139–151 (HSLGKWLGHPDKF) peptide in IFA (Difco Laboratories, Detroit, MI) with 800 μg *Mycobacterium tuberculosis hominis* H37Ra on days 0 and 7 (26). These inoculations were followed with 200 ng pertussis toxin i.p. (List Biological Laboratories) on days 0 and 2. The mice were followed for evidence of disease. Clinical severity was graded daily as follows: 0, no disease; 1, minimal or mild hind limb weakness (associated with limp tail); 2, moderate hind limb weakness or mild ataxia (waddling gait and/or poor righting ability); 3, moderate to severe hind limb weakness; 4, severe hind limb weakness or moderate ataxia; 5, paraplegia with no more than moderate four limb weakness; and 6, paraplegia with severe four-limb weakness or severe ataxia. A mean group clinical score was determined for each treatment arm.

### Immunoactive protein

Porcine ACTH (Ser-Tyr-Ser-Met-Glu-His-Phe-Arg-Trp-Gly-Lys-Pro-Val-Gly-Lys-Lys-Arg-Arg-Pro-Val-Lys-Val-Tyr-Pro-Asp-Ala [Gly]–Gly [Ala]-Glu-Asp-Gln-Ser [Leu]-Ala-Glu-Ala-Phe-Pro-Leu-Glu-Phe) was purchased from Bachem Pharmaceutical (Torrance, CA). Control peptide was scrambled ACTH (scrambled α-melanocyte-stimulating hormone [s-MSH]) (V-S-P-Y-K-S-G-M-W-E-R-H-F), an end-to-end mix of the first 13 aa of ACTH, the active moiety of ACTH 1–39 that interacts with the melanocortin receptor (27), generated by the Protein Chemistry Core Lab, Versiti Blood Research Institute.

### Dosing (feeding) regimen

Once immunized, mice that had attained a clinical score ~ 1 were randomized to one of two treatment groups and gavaged (fed) with 0.1 ml of 10 μg scrambled peptide (s-MSH) or 10 μg of ACTH daily using a 2.5-cm syringe fitted with a 22–24-gauge ball-point needle (Thomas Scientific, Swedesboro, NJ) as previously described (28).

### Isolation of lymphocytes from the LP.

Mice were euthanized by CO_2_ asphyxiation followed by cervical dislocation. The small intestine was harvested by cutting 2 cm below stomach and then carefully cutting away connective tissue for the first 5–10 cm. The small intestine was slowly pulled out of the abdomen, cut 1 cm above large intestine, and flushed with incomplete media (RPMI 1640; Life Technologies, Gaithersburg, MD) supplemented with 2% FBS (Gemini Bio Products, West Sacramento, CA) and 1% penicillin/streptomycin (Corning Life Sciences, Durham, NC) using an 18-gauge blunt needle and placed in fresh media (RPMI 1640: 5% FBS). Using a glass rod, the intestine was pulled up the rod, and the Peyer’s patches were removed. Forceps were then used to remove any fat on the bottom side of the intestine and sectioned into fifths. Using a cell scraper, intestinal pieces were scraped to remove residual mucous and feces left in the gut. The intestinal pieces were cut longitudinally and then into ~0.5–1-cm pieces and placed into a 15-ml conical tube with 10 ml of incomplete media, inverting the tube several times for cleansing. Intestinal pieces were placed into 20 ml of CMF solution (HBSS; Corning Life Sciences), 10 mM HEPES (Corning Life Sciences), 25 mM NaHCO_3_ (Thermo Fisher Scientific, Brookfield, WI), 1 mM EDTA (Thermo Fisher Scientific), 1 mM DTT (Sigma-Aldrich, Milwaukee, WI), and 2% FBS (R&D Systems, Minneapolis, MN), shaken for 30 min at 37°C, and then vortexed for 15 s and wash repeated. EDTA was removed by shaking pieces in complete media for 5 min. Intestinal pieces were added to 10 ml of digestion media (RPMI 1640; 10% FBS and 1 mg/ml collagenase D [Roche/Sigma-Aldrich, Milwaukee, WI]), 0.4 mg/ml dispase (STEMCELL Technologies, Cambridge, MA), and 0.1 mg/ml DNase I (Sigma-Aldrich), shaken for 1.5 h at 37°C at 250–300 rpm, vortexed for 15 s, filtered through a cell strainer, and rinsed with 5 ml of complete media. Cells were spun and resuspended in 8 ml of 44% Percoll (GE Healthcare, Marlborough, MA) (1× stock solution: 10× PBS and Percoll diluted to 44% with RPMI 1640). Five milliliters of 67% Percoll was added to FBS-coated tubes and 44% Percoll was carefully layered over the 67%, which was spun at 1620 rpm for 20 min. The top layer of the gradient was carefully removed, and then the cell layer was removed, placed in a fresh tube, and diluted with complete media. Cells were spun down, supernatant removed, washed one more time, and cells counted.

### Isolation of splenocytes and CNS lymphocytes

The mouse was sprayed with 70% ethyl alcohol (ETOH) on the left flank, and scissors and tweezers were placed in ETOH for sanitization. The skin was pinched just below the rib cage with tweezers, and a small incision was made at a downward 45° angle. The skin was pulled apart with fingers at the site of the incision, the inner membrane was snipped to expose the body cavity, and then the spleen was isolated and placed in 5-ml of media.

### Splenocyte cell isolation.

Under sterile conditions, media and the spleen were placed in a petri dish. Using frosted glass slides, the spleen was crushed between the rough side of the slides, and the slides were washed off using 5 ml of fresh media and media placed in a fresh tube along with a 5-ml rinse of the petri dish. The cells were spun down at 1200 rpm for 5 min. The media was decanted, cells were resuspended, and 900 μl of distilled H_2_O was added gently; the cells were agitated by flicking, then 10 ml of 10× PBS was added to generate 1× PBS, and 10 ml media was added. Cells were spun down for 5 min at 1,200 rpm and media decanted. Cells were resuspended in 10 ml of media, the debris allowed to settle to the bottom, and then the media poured off with the cells placed into new tube. Cell viability and count were determined using Trypan blue.

### CNS cell isolation.

The mouse was anesthetized, and the abdomen, wetted with 70% ETOH, was opened to expose the heart. A cut in the right atrium was made and then a 27-gauge needle attached to a 50-ml syringe; the animal was perfused with ~30–50 ml of ice-cold PBS, allowing the lungs and liver to lose color. The animal’s inner organs were removed by picking up the bladder and the intestines and cutting them all away, up to the lungs and the heart. Then spinal cord was cut just above the hips, the animal turned over, and the head cut off using scissors. The skin on the skull was removed, and using forceps, the skull was gripped in the eye sockets, and a cut was made where then brain meets the spinal cord along the left side of the skull. The skull was peeled back from the incision exposing the brain and the brain scooped out into ice-cold PBS. The spinal column was removed by cutting through the ribs and using a blunt 18-gauge needle pushed through the bottom of the spinal cord, and the spinal canal was flushed out and the cord placed into ice-cold PBS. Up to one brain and a spinal cord were placed in = ml of PBS into a glass tissue homogenizer, and the tissue was repeatedly crushed until completely homogenized. Using fresh PBS, the plunger was rinsed off, and the homogenate was strained through a 70-μm nylon cell strainer over a 50-ml conical tube, producing a single-cell suspension. The cells were spun at 2000 rpm for 5 to 6 min. The liquid was aspirated and cells resuspended in 8 ml of 40% Percoll. Three milliliters of 80% Percoll was added into precoated 15-ml Falcon tubes with FBS, and a layer of 40% Percoll cell suspension was placed on top of 80% and spun at 1500 rpm for 30 min without braking. The lipid layer was aspirated as well as almost all of the 40% Percoll as the cells are suspended between the two Percoll layers. The interface was removed to a new tube and filled with media, spun, the media aspirated off the cells, 10 ml of RPMI 1640 with L-glutamine added, mixed, and washed. Thereafter, the appropriate amount of media was added to the cells and the cells counted.

### Flow cytometry

Lymphocytes were all stained with the anti-mouse Abs CD4 PE-F594 (BD Biosciences, San Jose, CA), CD4 FITC (BioLegend, San Diego, CA), CD11b AF-700 (BioLegend), and GL3 (γδ TCR). All cytokine staining was performed using the BD Foxp3 intranuclear transcription factor staining kit for Foxp3 PE (eBioscience), T-bet PE (BioLegend), IL-17a Rat PE-Cy7 (BioLegend), IL-17a Alexa Fluor 488 (BD Biosciences), IFN-γ PerCP-Cy5.5 (BD Biosciences), IFN-γ allophycocyanin-Cy7 (BioLegend), IL-10 allophycocyanin (BioLegend), and IL-6 allophycocyanin (BioLegend) following the manufacturer’s instructions. For phenotypic analysis, flow cytometry was performed using a BD Biosciences LSR II flow cytometer, and analysis was performed using FlowJo (Tree Star, Ashland, OR).

### Statistics

Statistical analysis was performed using unpaired *t* test (Prism 9.0; GraphPad, San Diego, CA).

## RESULTS

### Oral ACTH attenuates EAE disease severity

**FIGURE 1.**
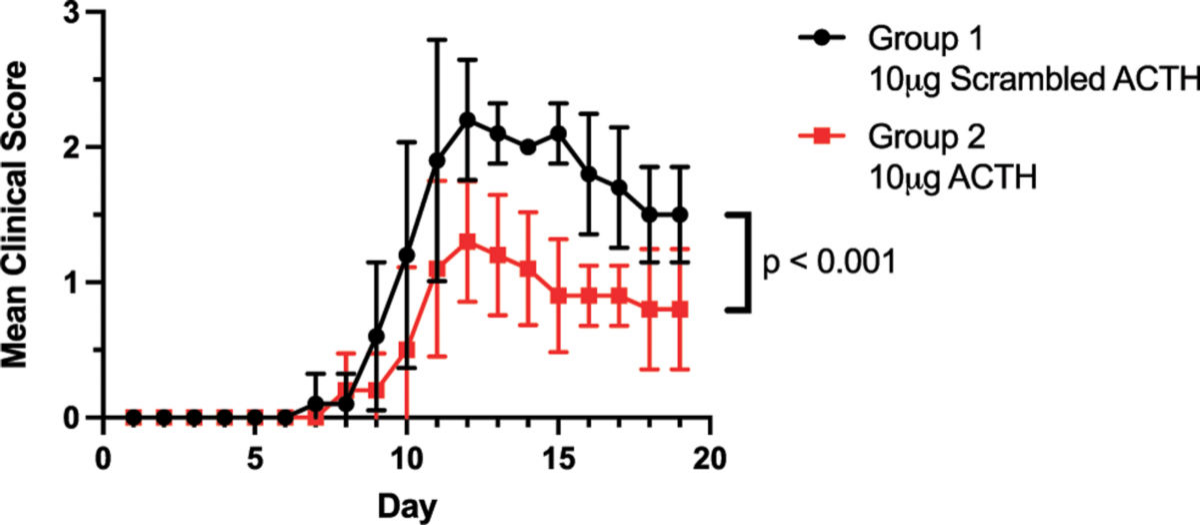
Oral ACTH attenuated EAE severity in the SJL/J mice. SJL/J mice (*n* = 5/group) were immunized with PLP peptide 139–151 and gavaged daily with 0.1 ml of control scrambled peptide (s-MSH) or 10 μg ACTH as described in *Materials and Methods* (unpaired *t* test, *p* < 0.001, from day 10–19, average EAE score ± SEM). The data are representative of three separate experiments (total *n* = 15/group).

We examined the MOA of ingested (orally administered) 10 μg ACTH in EAE in the GALT, spleen, and CNS in SJL/J mice. Mice were immunized and separated into two groups, when each mouse attained a clinical score of 1 (day 10 postimmunization), at which time oral dosing was initiated. The 10 μg s-MSH (scrambled) group reached a mean group clinical score of 2.2 ± 0.3 12 d postimmunization (Fig. 1). In addition, the ACTH-treated group exhibited reduced signs of EAE throughout the entire disease curve as compared with the scrambled control group (Fig. 1). In contrast, the active ACTH treatment group reached an average maximum clinical score of 1.2 ± 0.3 on day 12 (Fig. 1). These data indicate that ingested 10 μg ACTH attenuated EAE disease severity in SJL/J mice (*p* < 0.001) (Fig. 1).

### Oral ACTH upregulates anti-inflammatory responses in the LP during EAE

**FIGURE 2.**
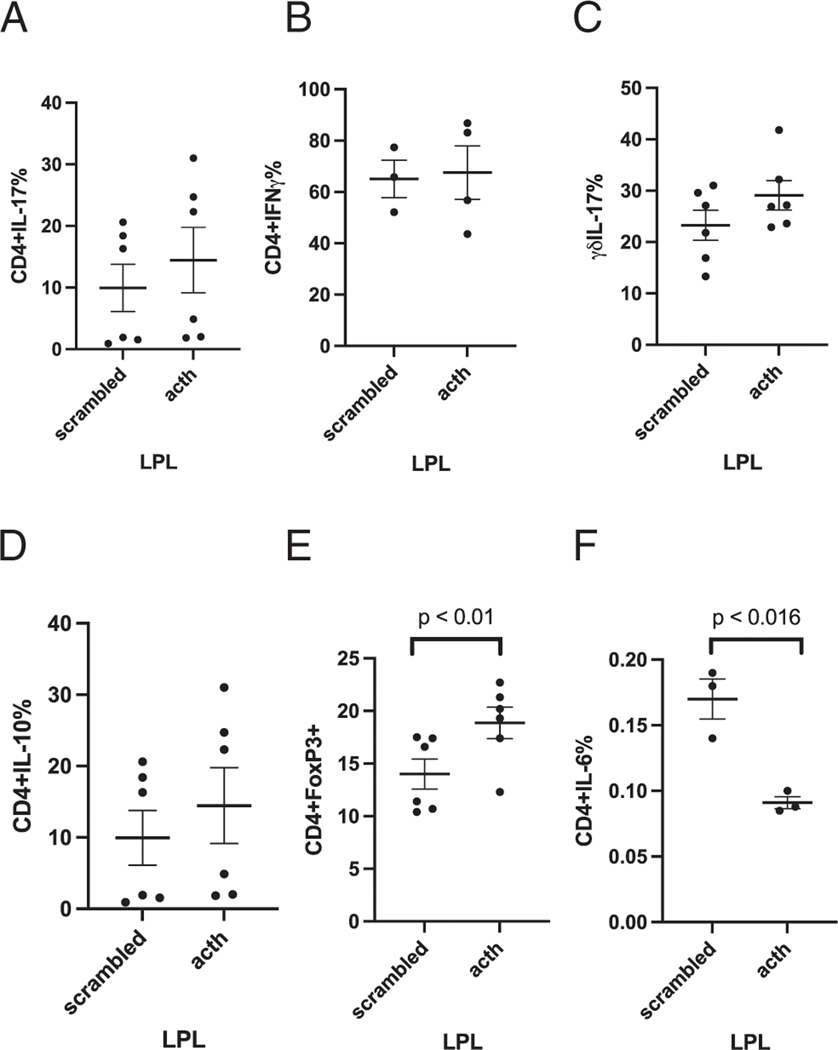
Oral ACTH increased the frequency of CD4^+^IL-10^+^– producing cells and T_reg_ cells and decreased CD4^+^IL-6^+^–producing cells in the LP. Lymphocytes were stained as described in *Materials and Methods*. Results are expressed as percentage of cells positive for both CD4^+^ IL-17^+^ (**A**), CD4^+^IFN-γ (**B**), δγIL-17^+^ (**C**), CD4^+^IL-10^+^ (**D**), T_reg_ cells (**E**), and CD4^+^IL-6^+^ (**F**). The data are representative of three separate experiments (mean ± SEM). Each data point represents one mouse.

**FIGURE 3.**
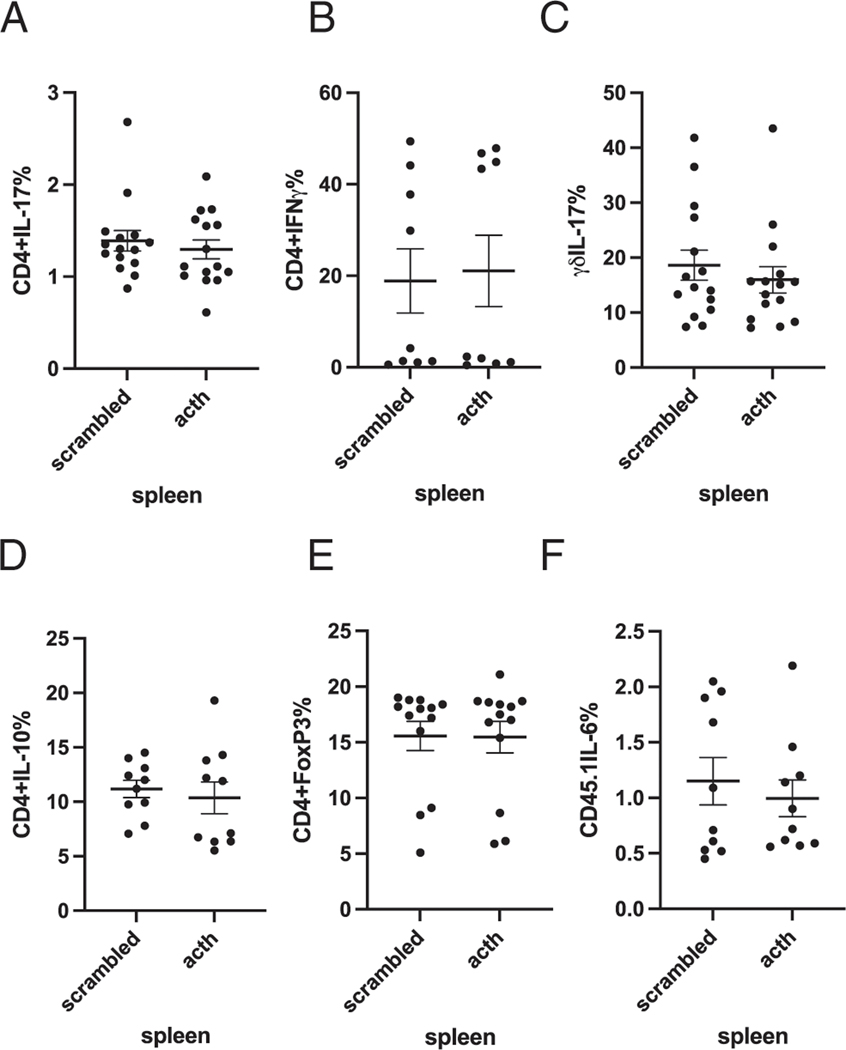
Oral ACTH did not have a significant effect on the frequencies of CD4^+^IL-17^+^, CD4^+^IL-10^+^, CD4^+^IL-6^+^, CD4^+^IFN-γ, or δγIL-17^+^ production or the percentage of T_reg_ cells in the spleen. Results are expressed as percentage of cells positive for both CD4^+^ IL-17^+^ (**A**), CD4^+^IFN-γ (**B**), δγIL-17^+^ (**C**), CD4^+^IL-10^1^ (**D**), T_reg_ cells (**E**), and CD4^+^IL-6^+^ (**F**). This experiment represents results from three separate experiments (mean ± SEM). Each data point represents one mouse.

**FIGURE 4.**
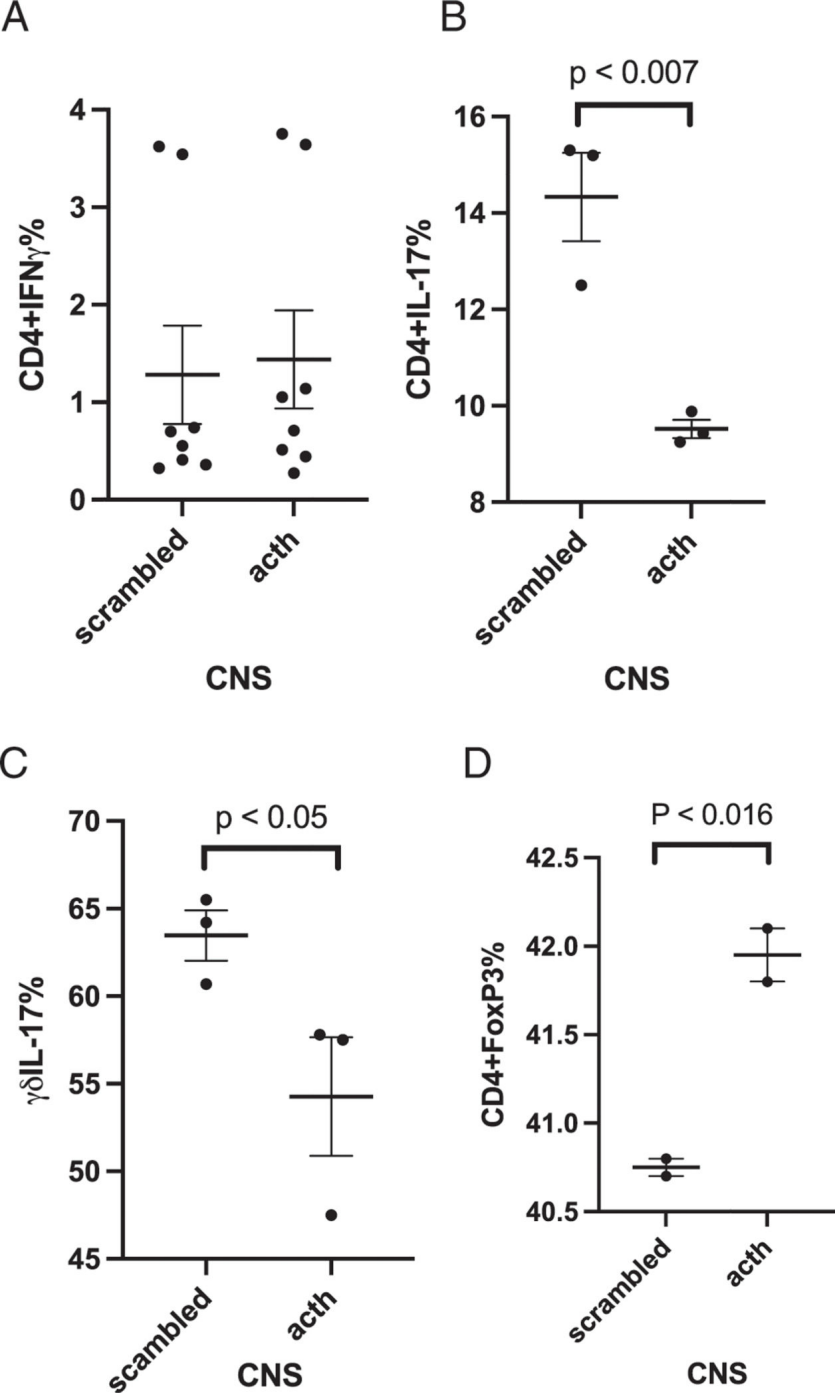
Oral ACTH decreased the frequency of CD4^+^ and δγIL-17^+^–producing T cells in the CNS while increasing the frequency of T_reg_ cells. Results are expressed as percentage of cells positive for CD4^+^IFN-γ (**A**), CD4^+^IL-17^+^ (**B**), γδ IL-17^+^ (**C**), and T_reg_ cells (**D**). The data are representative of two separate experiments (mean ± SEM). Each data point represents one mouse.

**FIGURE 5.**
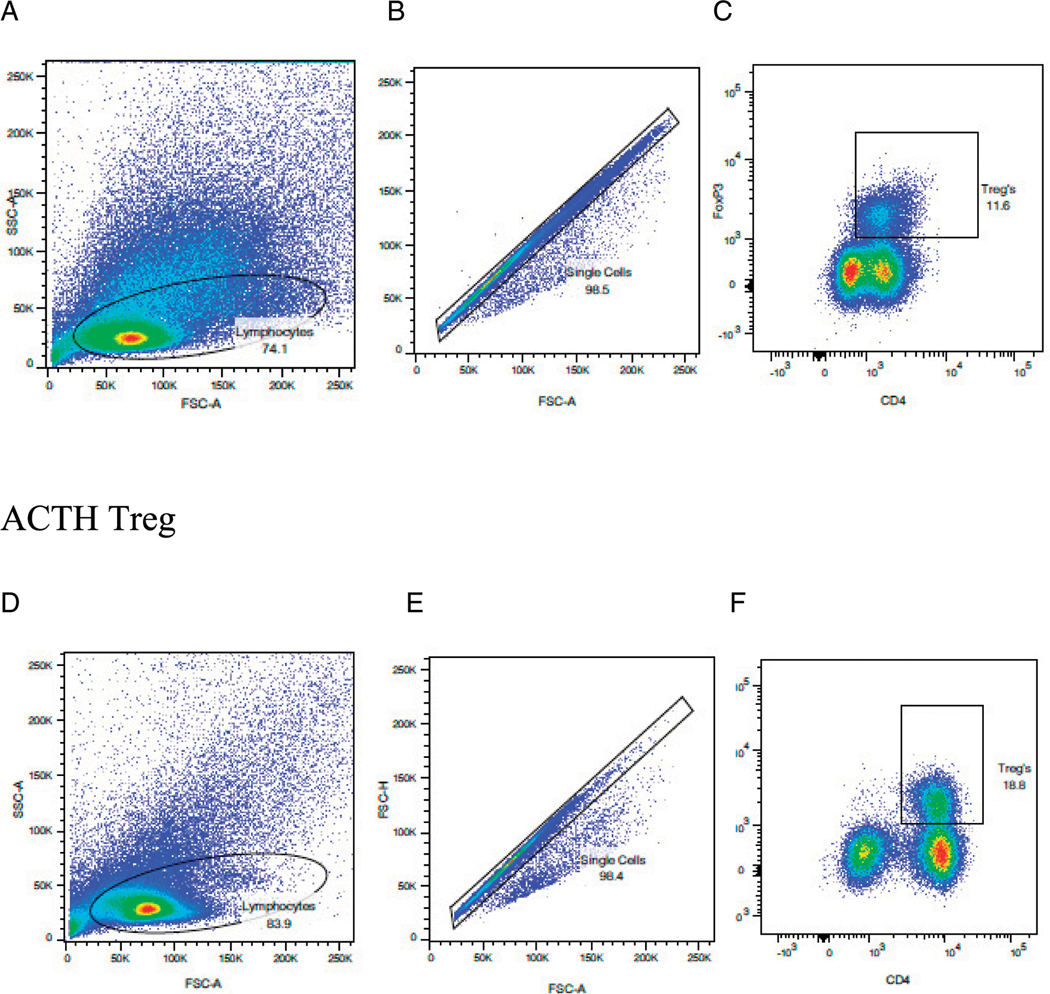
Oral ACTH increased the frequency of T_reg_ cells in the LP. SJL/J mice were immunized with PLP peptide 139–151 and gavaged daily with 0.1 ml of control scrambled peptide (s-MSH) or 10 μg ACTH as described in *Materials and Methods* and Fig. 2. Side scatter/forward scatter, singlets, and cytokine staining are shown for s-MSH (**A–C**) and ACTH (**D–F**). Lymphocytes were parent gated from the experiments outlined above, obtained on day 19, and stained as described in Fig. 2. Results are expressed as percentage of cells positive for T_reg_ and representative of three separate experiments.

**FIGURE 6.**
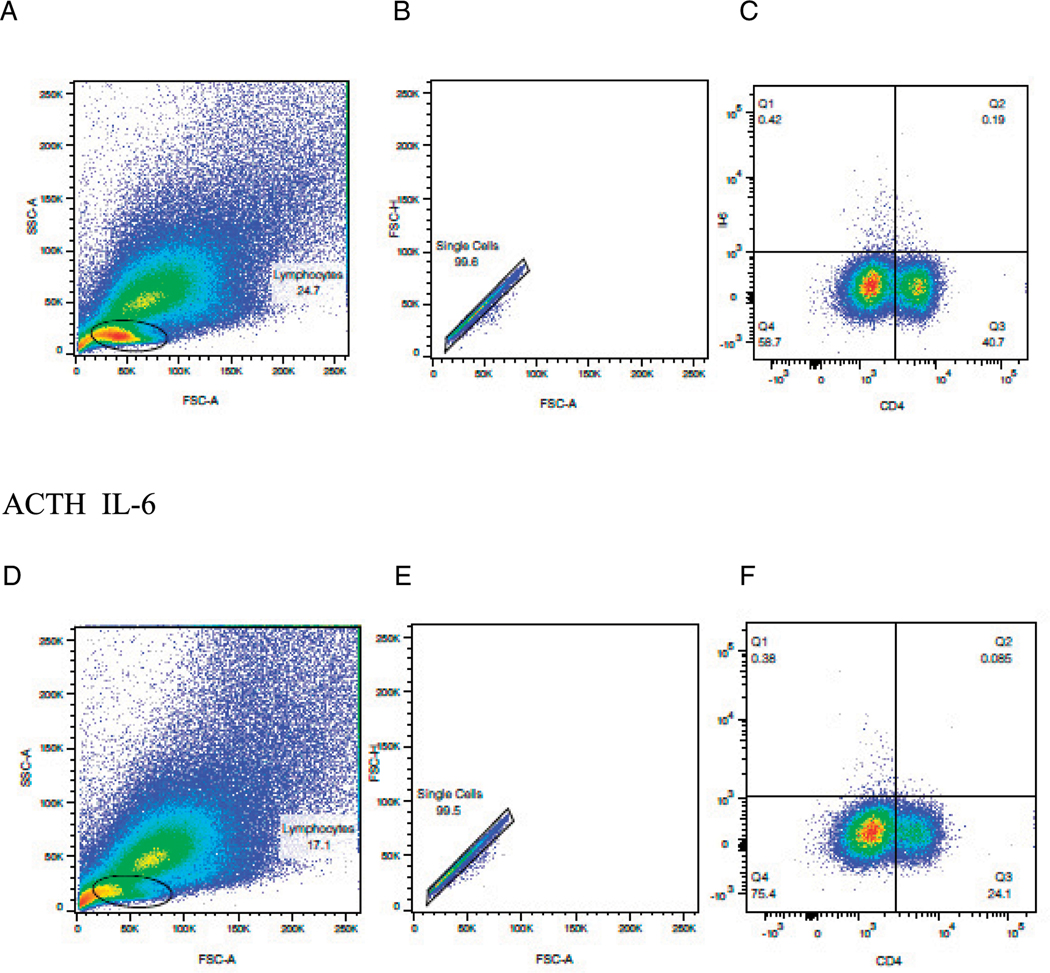
Oral ACTH decreased CD4^+^IL-6^+^–producing cells in the LP. SJL/J mice were immunized with PLP peptide 139–151 and gavaged daily with 0.1 ml of control scrambled peptide (s-MSH) or 10 μg ACTH as described in *Materials and Methods* and Fig. 2 above. Side scatter/forward scatter, singlets, and cytokine staining are shown for s-MSH (**A–C**) and ACTH (**D–F**). Lymphocytes were parent gated from the experiments outlined above, obtained on day 19, and stained as described in Fig. 2. Results are expressed as percentage of cells positive for CD4^+^IL-6^+^ and representative of three separate experiments.

We next examined the effect of oral ACTH on the frequency of IL-17, IL-10, IL-6, T_reg_ cells, and IFN-γ-producing cells in different immune compartments during EAE. In the LP, there was no difference in the percentage of CD4^+^IL-17^+^ T cells (Fig. 2A), CD4^+^IFN-γ^+^ (Fig. 2B), IL-17^+^ γδ (Fig. 2C), or CD4^+^IL-10–producing cells (Fig. 2D) in ACTH versus s-MSH (scrambled)–fed mice. Consistently, T_reg_ cells were increased in the LP in ACTH-treated mice compared with LP from mice fed the scrambled peptide (*p* < 0.01) (Fig. 2E). Of interest, proinflammatory CD4^+^IL-6^+^–producing T cells were decreased in frequency in the LP of ACTH-treated as compared with the scrambled control (*p* < 0.016) (Figs. 2F, compare to Fig. 3 [spleen] and Fig. 4 [CNS]). The lymphocyte parent gating, singlets, and cytokine staining are shown in Fig. 5 for T_reg_ cells and Fig. 6 for IL-6. These data indicate that ACTH, although having no effect on proinflammatory (Th17 and Th1) cells, upregulates an anti-inflammatory program (T_reg_), including a decrease in the frequency of T cells producing proinflammatory cytokine IL-6.

### Oral ACTH does not alter pro- and anti-inflammatory programs in the spleen during EAE

To determine whether ACTH altered immune profiles outside of the gut, the frequency of inflammatory and counterregulatory-producing T cell and T_reg_ cell subsets affected by oral ACTH treatment was examined in the spleen. There were no significant changes in CD4^+^IL-17^+^, CD4^+^IFN-γ^1^, γδ IL-17^+^, CD4^+^IL-10, T_reg_, or CD4^+^IL-6^+^ T cells in the spleen (Fig. 3A–F, respectively). These data indicate that oral ACTH does not exhibit a systemic effect on the peripheral immune system.

### Oral ACTH reduced expression of IL-17–*producing T cells in the CNS during EAE*

**FIGURE 7.**
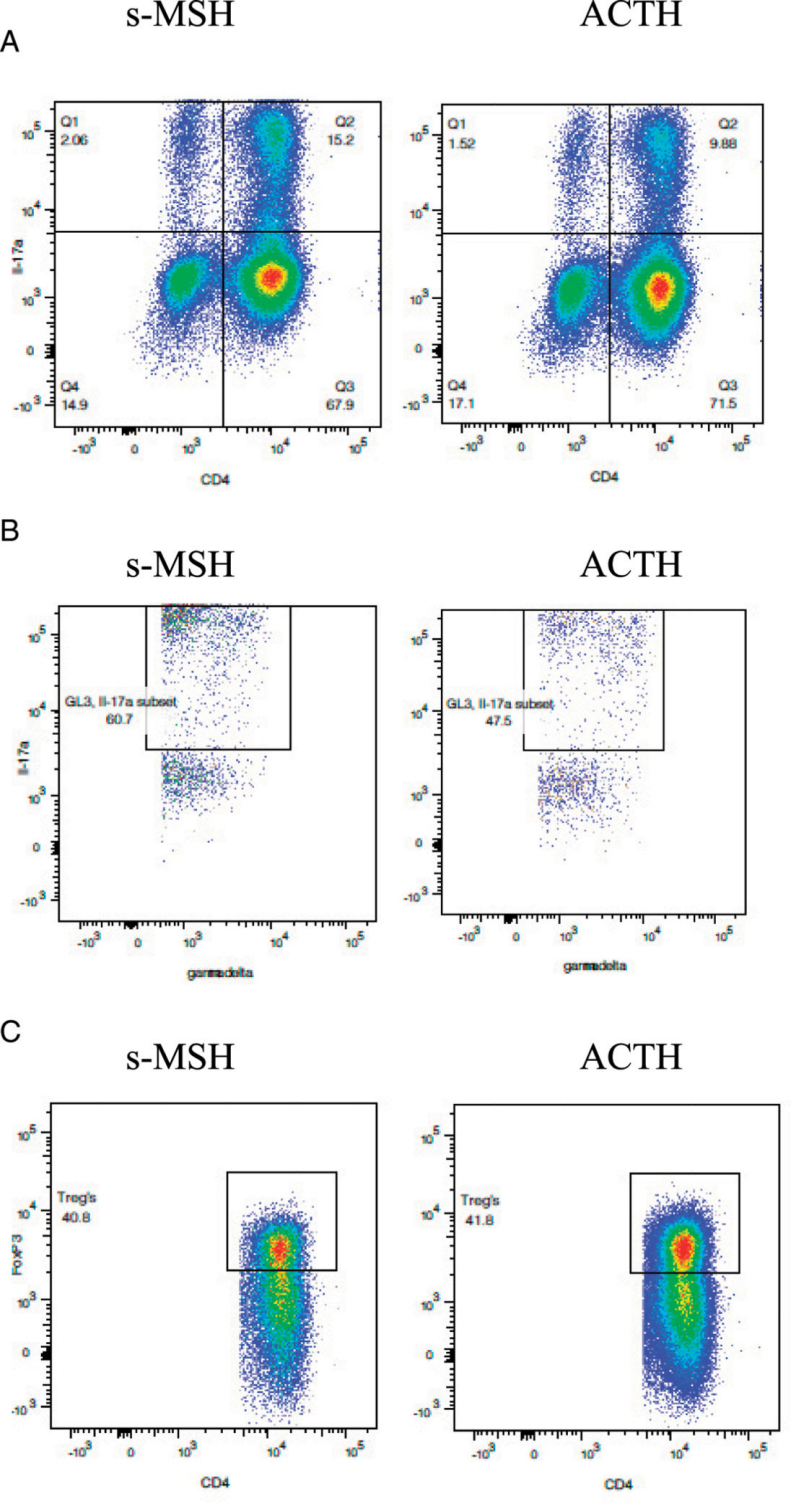
Oral ACTH decreased the frequency of CD4^+^ and γδIL-17^+^–producing T cells in the CNS while increasing the frequency of T_reg_ cells. SJL/J mice were immunized with PLP peptide 139–151 and gavaged daily with 0.1 ml of control scrambled peptide (s-MSH) or 10 μg ACTH as described in *Materials and Methods* and Fig. 4. Lymphocytes were parent gated from the experiments outlined above, obtained on day 19, and stained as described in Fig. 4. Results are expressed as percentage of cells positive for CD4^+^IL-17^+^ (**A**), γδIL-17^+^ (**B**), and T_reg_ cells (**C**). The data are representative of two separate experiments.

Because EAE severity was reduced in ACTH-treated mice, we determine whether the balance of pro- and anti-inflammatory cells was altered within the CNS. Although there were no significant changes of CD4^+^IFN-γ-producing cells in the CNS (Fig. 4A) (*p* < 0.86), there was a significant decreased frequency of effector CD4^+^IL-17^+^–producing T cells (Fig. 4B) (*p* < 0.026) and δγ IL-17^+^ T cells (Fig. 4C) (*p* < 0.046). There was a slight but significant increase in the frequency of T_reg_ cells in the CNS comparing ACTH-fed mice to s-MSH–fed mice (Fig. 4D) (*p* < 0.016). IL-6 and IL-10 were unable to be determined in the CNS due to limited cell sampling. Cytokine staining is shown in Fig. 7.

## DISCUSSION

Despite the regulatory role of IL-17–producing cells providing immunity at mucosal surfaces as a first line of defense against infection (29, 30), our data in this inflammatory model show an overall anti-inflammatory effect of ingested ACTH on IL-17 in the SJL/J strain of EAE-susceptible mice in the LP and CNS. Most importantly, there was a significant decrease in frequency of CD4^+^ and δγ T cells producing in the CNS of mice fed ACTH compared with mice fed control s-MSH, along with a small but significant T_reg_ cell increased frequency. There was an increase in the frequency of CD4^+^IL-10^+^ T cells in the LP in ACTH-treated mice compared with mice fed control s-MSH. Although low in overall frequency, there were also decreased CD4^+^IL-6^+^ T cells in the LP in ACTH-treated mice compared with mice fed control s-MSH. The anti-inflammatory effect presumably started in the LP after ACTH feeding, resulting in increased frequencies of CD4^+^IL-10–producing T cells and T_reg_ cells in the ACTH-fed group. Although Th17 cells are encephalitogenic, they typically lose IL-17 expression in the CNS and secrete IFN-γ (14). In contrast, in this study, EAE seems to be driven by IL-17, as suggested by Hofstetter and Forsthuber (12) and Kroenke et al. (13). We also showed a decrease in the frequency of γδ T cells producing IL-17 in the CNS during EAE. In our present model, IL-17–producing γδ T cells may play a significant role in CNS disease. Although some have suggested that γδ T cells may facilitate recovery in EAE (31), others have found that γδ T cells contribute to encephalogenicity (32, 33).

We saw similar but not identical findings in the SJL/J and B6 strains from previous work. In previous EAE studies using B6 mice ingesting oral ACTH (21), ingested (oral) ACTH inhibited ongoing clinical disease. In the B6 mice, there were significantly less CD11b^+^IL-6^+^– and CD4^+^IL-17^+^–producing lymphocytes from ACTH-fed mice compared with s-MSH–fed mice in the LP. There was also a decrease in the frequency of IL-17– and IFN-γ–producing splenic cells and an increase in T_reg_ cell frequency in ACTH-fed mice compared with s-MSH-fed control spleens. There were less CD4^+^ IFN-γ-producing CNS lymphocytes in ACTH-fed mice compared with s-MSH–fed control CNS.

Using the SJL/J mice, we showed decreased levels of IL-17 as a potential mechanism of protection against clinical disease in the CNS. Overall, it appears that there are some common features to the MOAs of oral ACTH in different strains of EAE-susceptible mice. These include decreased IL-17 and increased T_reg_ cells after oral ACTH in one or more immune compartments.

Others have shown previously that parenteral α-MSH, the active moiety of ACTH containing the first 13 aa of ACTH, can inhibit EAE (34). ACTH can increase IL-10 production in human cells (35), human PBMCs (36), and mouse splenocytes (37), resulting in IL-10 that could inhibit CD4^+^ T cell IL-17 production (38). Even though we could not determine that there were increased IL-10 levels in the CNS, increased IL-10 production after ACTH feeding in the GALT could provide an anti-inflammatory signal reducing IL-17 production outside the LP. ACTH could also promote the formation of T_reg_ cells by increasing the expression of IL-2Rα (CD25) in CD4^+^ T cells and activation of STAT5 in CD4^+^CD25^+^ T cells (39–41). In SJL mice, we found a similar T_reg_ cell effect in the GALT at the location of first contact with ACTH that continued in the CNS.

Therapeutic strategies for MS exacerbations since the 1950s have been based on the use of corticotropin hormone (ACTH) that supposedly released endogenous corticosteroids. Parenteral corticosteroids reduce the inflammatory responses by inhibiting lymphocyte proliferation and cell-mediated immune response, downregulating cytokine gene expression, and have independent effects on blood–brain barrier permeability (42, 43).

However, corticotropin’s major therapeutic effects are more likely of direct immunomodulatory effects via melanocortin receptors (MCR). MC peptides are pro-opiomelanocortin–derived amino acid sequences, including ACTH (corticotropin) and α-MSH (44). After oral administration, immunoactive proteins such as ACTH are probably not absorbed. However, lymphocytes in the LP are situated just beneath a single layer of epithelial cells that include CD4^+^ and other T cells (45). Therefore, lymphocytes in the LP have direct contact with polypeptides in the intestinal lumen. ACTH, particularly in the proximal small bowel, would have access to lymphocytes and their MCR (45, 46). ACTH, after surviving passage through the upper gastrointestinal tract, most likely inhibits lymphocyte activation directly via MCR (melanocortin effect) in the GALT that is unrelated to stimulation of glucocorticoid release (corticotropin effect) (47, 48). This is where gavaged ACTH would be primarily deposited in our experiments (personal observation using methylene blue gavage). ACTH can bind to specific MCR: MC-3R (melanocortin receptor-3) that can stimulate an anti-inflammatory effect (49, 50) and MC5r (melanocortin receptor-5), both of which are partially responsible for induction of T_reg_ cells (40). Once LP lymphocytes are activated via MCR by ACTH, they can migrate to the mesenteric lymph nodes and beyond systemically to organs undergoing inflammatory responses, such as, in our case, the CNS (51).

The administration of endogenous immunoactive proteins via the gut offers an alternative to systemic application (23), with ease of administration in chronic clinical use (52) and patient convenience (53), and would be a great step forward because gut delivery is easy, well tolerated, and inexpensive with a favorable therapeutic index (54, 55). By establishing that oral ACTH can inhibit EAE in two mouse strains, this supports pursuing additional clinical trials of ingested (oral) ACTH in subjects with MS.

## Abbreviations used in this article:

ACTH: adrenocorticotropic hormone
B6: C57BL/6
EAE: experimental autoimmune encephalomyelitis
ETOH: ethyl alcohol
GALT: gut-associated lymphoid tissue
LP: lamina propria
MCR: melanocortin receptor
MOA: mechanism of action
MS: multiple sclerosis
PLP: proteolipid protein
s-MSH: scrambled α-melanocyte-stimulating hormone
